# Redundant Function of REV-ERBα and β and Non-Essential Role for Bmal1 Cycling in Transcriptional Regulation of Intracellular Circadian Rhythms

**DOI:** 10.1371/journal.pgen.1000023

**Published:** 2008-02-29

**Authors:** Andrew C. Liu, Hien G. Tran, Eric E. Zhang, Aaron A. Priest, David K. Welsh, Steve A. Kay

**Affiliations:** 1 Section of Cell and Developmental Biology, Division of Biological Sciences, University of California San Diego, La Jolla, California, United States of America; 2 Genomics Institute, Novartis Research Foundation, San Diego, California, United States of America; 3 Department of Psychiatry, University of California San Diego, La Jolla, California, United States of America; 4 Veterans Affairs, San Diego Healthcare System, San Diego, California, United States of America; Howard Hughes Medical Institute, Northwestern University, United States of America

## Abstract

The mammalian circadian clockwork is composed of a core PER/CRY feedback loop and additional interlocking loops. In particular, the ROR/REV/*Bmal1* loop, consisting of ROR activators and REV-ERB repressors that regulate *Bmal1* expression, is thought to “stabilize” core clock function. However, due to functional redundancy and pleiotropic effects of gene deletions, the role of the ROR/REV/*Bmal1* loop has not been accurately defined. In this study, we examined cell-autonomous circadian oscillations using combined gene knockout and RNA interference and demonstrated that REV-ERBα and β are functionally redundant and are required for rhythmic *Bmal1* expression. In contrast, the RORs contribute to *Bmal1* amplitude but are dispensable for *Bmal1* rhythm. We provide direct in vivo genetic evidence that the REV-ERBs also participate in combinatorial regulation of *Cry1* and *Rorc* expression, leading to their phase-delay relative to *Rev-erbα*. Thus, the REV-ERBs play a more prominent role than the RORs in the basic clock mechanism. The cellular genetic approach permitted testing of the robustness of the intracellular core clock function. We showed that cells deficient in both REV-ERBα and β function, or those expressing constitutive BMAL1, were still able to generate and maintain normal *Per2* rhythmicity. Our findings thus underscore the resilience of the intracellular clock mechanism and provide important insights into the transcriptional topologies underlying the circadian clock. Since REV-ERB function and *Bmal1* mRNA/protein cycling are not necessary for basic clock function, we propose that the major role of the ROR/REV/*Bmal1* loop and its constituents is to control rhythmic transcription of clock output genes.

## Author Summary

Circadian clocks in plants, fungi, insects, and mammals all share a common transcriptional network architecture. At the cellular level, the mammalian clockwork consists of a core *Per/Cry* negative feedback loop and additional interlocking loops. We wished to address experimentally the contribution of the interlocking *Bmal1* loop to clock function in mammals. Because behavioral rhythms do not always reflect cell-autonomous phenotypes and are subject to pleiotropic effects, we employed cell-based genetic approaches and monitored rhythms longitudinally using bioluminescent reporters of clock gene expression. We showed that REV-ERB repressors play a more prominent role than ROR activators in regulating the *Bmal1* rhythm. However, significant rhythmicity remains even with constitutive expression of *Bmal1*, pointing to the resilience of the core loop to perturbations of the *Bmal1* loop. We conclude that while the interlocking loop contributes to fine-tuning of the core loop, its primary function is to provide discrete waveforms of clock gene expression for control of local physiology. This study has important general implications not only for circadian biology across species, but also for the emerging field of systems biology that seeks to understand complex interactions in genetic networks.

## Introduction

Circadian rhythms in physiology and behavior are regulated by endogenous circadian clocks. All the molecular clocks so far described in multicellular organisms constitute negative feedback loops in which protein products of clock genes inhibit transcription of their own genes [1]. In mammals, the central pacemaker in the suprachiasmatic nuclei (SCN) integrates light-dark cycle input and coordinates oscillators in peripheral tissues [2]. Like the SCN, peripheral tissues also contain cell-autonomous circadian oscillators. The current cellular clock model comprises a core feedback loop consisting of PER and CRY repressors and BMAL1 and CLOCK activators [1],[3]. In the core loop, BMAL1/CLOCK heterodimers directly bind to E-box enhancer elements present in *Per* (*Per1* and *Per2*) and *Cry* (*Cry1* and *Cry2*) genes and activate their transcription; PER and CRY proteins in turn repress their own transcription through direct interactions with BMAL1/CLOCK.

The mammalian clock has been shown to contain additional interlocking loops. In particular, the ROR/REV/*Bmal1* feedback loop consists of the RORs (RORa, b and c) and REV-ERBs (REV-ERBα and β), members of a subfamily of orphan nuclear receptors [4], whose expression is directly regulated by the core loop [5]–[8]. To drive rhythmic expression of *Bmal1*, REV-ERBα represses *Bmal1* transcription by directly binding to the ROR elements (ROREs) in the *Bmal1* promoter [5],[9]; RORa and RORb, on the other hand, act as positive drivers to activate *Bmal1* expression in the SCN [6], [9]–[11]. The roles of REV-ERBβ and RORc in clock function have not been addressed.

An analogous set of interlocking loops has been described in the *Drosophila* circadian clock [7],[12],[13]. The dPER/dTIM repressors and dCLK/dCYC activators constitute the core feedback loop. In the interlocked *dClk* feedback loop, the bZIP transcription factors dPDP1 and dVRI, which are directly controlled by the core loop, activate and repress *dClk* transcription, respectively. However, unlike the requirement for cyclic expression of *dPer* and *dTim* mRNAs, it was shown that *dClk* mRNA cycling is not necessary for molecular and behavioral rhythms in flies [14]–[16]. The *dClk* loop function in flies could not be precisely defined genetically, however, because mutants deficient in *dVri* and/or *dPdp* are developmentally lethal [12],[16].

The role of the ROR/REV/*Bmal1* loop in mammals has not been precisely addressed either, due to functional redundancy of the RORs and REV-ERBs and pleiotropic effects of gene deletions. As deletion of *Rev-erbα*, *Rora* or *Rorb* results in a broader distribution of circadian period lengths, it was suggested that the ROR/REV/*Bmal1* loop serves as a stabilizing mechanism [5]–[7],[10]. However, *Ror* mutant mice exhibit potentially confounding non-circadian phenotypes. *Rorb* ^−/−^ mice display reproductive deficits and a severe postnatal retinal degeneration [10]. *Rora* knockout (*Rora^−/−^*) and mutant *staggerer* (*Rora^sg/sg^*) mice display cerebellar ataxia and are mostly infertile [6],[11],[17],[18]. Importantly, period dispersion is not unique to animals deficient in *Ror* or *Rev-erb* function; *Per1^−/−^*, *Per2* ^−/−^ and *Clock^m/m^* mice also display less persistent circadian behavior and larger variability of periods [19]–[22]. Thus, circadian abnormalities in these mice measured using behavioral outputs may not faithfully reflect intracellular clock function. Finally, functional redundancy cannot be addressed genetically at the behavioral level because compound knockout animals have gross defects.

The drawbacks of behavioral analysis can be circumvented by studies using cell-based clock models. Strategically, molecular mechanisms required for rhythmicity are best studied at the cellular level using long-term recordings to assess persistence of circadian rhythmicity [23]. In this study, by taking advantage of a cell-based experimental model and real-time bioluminescence monitoring of gene expression, we first define the roles of RORc and REV-ERBβ in peripheral clock function, and then extend our studies to include all the RORs and REV-ERBs and their respective contributions to circadian rhythms of *Bmal1* expression. Furthermore, we show that the REV-ERBs are necessary for *Bmal1* rhythm while the RORs are dispensable, indicating that the REV-ERBs play a more prominent role than the RORs in the transcriptional circuitry of the clockwork. Importantly, however, rhythmic *Bmal1* mRNA and protein expression is not required for the basic operation of the core clock. These results are in line with the observation that constitutive *Bmal1* expression was able to rescue circadian behavioral rhythms in *Bmal1^−/−^* mice [24]. We suggest that the major role of the ROR/REV/*Bmal1* loop is to provide additional phase modulation for establishing transcriptional output networks.

## Results

### Differential Tissue Distribution of the Rors

We first examined expression of the *Rors* and *Rev-erbs* in various tissues (Figure S1A, S1B, S1C). In contrast to the ubiquitous expression of *Bmal1*, *Rev-erbα* and *Rev-erbβ* in all the tissues examined, expression and rhythmicity of the RORs are more restrictive. *Rora* expression is ubiquitous, but its circadian cycling is restricted to SCN. *Rorb* is expressed in the SCN, hypothalamus, cerebral cortex and retina, but not in the liver. Conversely, *Rorc* is rhythmically expressed in the liver, but not detected in the SCN or other brain regions. Expression patterns of the *Ror* genes in the lung were similar to those in the liver (data not shown). The tissue-specific expression patterns of the RORs are consistent with previous reports [6], [25]–[28].

In this study, we extensively used fibroblasts derived from mice as a cell-based clock model. Of the three *Rors*, only *Rora* is highly expressed in mouse fibroblasts, but no distinct mRNA rhythm was detected (Figure S1A); *Rorb* and *Rorc* were not detected in fibroblasts (Figure S1A and S1B). Differential tissue distribution and rhythmicity of the *Rors* suggests that they may have different functions in clock mechanisms.

### Rorc^−/−^ Mice Display Normal Circadian Rhythms

*Rora* and *Rorb* have been characterized as clock components, functioning to regulate *Bmal1* expression in the SCN (Figure S1C) [6],[10],[11],[25]. However, since RORc is not expressed in the SCN, it should not affect function of the SCN pacemaker, which drives circadian locomotor behavior. We tested this hypothesis in a mouse line deficient in *Rorc* function. Deletion of *Rorc* results in reduced survival of thymocytes and abnormal lymphoid organ development, but *Bcl-xL* transgene (*Bcl-xL^Tg^*) expression restored most aspects of normal thymocyte development and significantly improved animal survival [29]. Compared to *Bcl-xL^Tg^* control (period length τ = 23.42 hr±0.08, n = 5), *Rorc^−/−^:Bcl-xL^Tg^* mice displayed normal circadian wheel-running activity under constant darkness (τ = 23.34 hr±0.2, n = 8). These mice also showed a normal response to a light pulse at CT16 (Figure S1D). We further examined the dynamics of molecular rhythms in the SCN and showed that SCN explants from *Rorc^−/−^:Bcl-xL^Tg^* mice also displayed similar *mPer2^Luc^* bioluminescence rhythms to control mice (data not shown). Thus, consistent with the absence of *Rorc* gene expression in the SCN, these results confirm that RORc plays no role in SCN pacemaker function.

### RORc Regulates Circadian Bmal1 Transcription in the Liver

**Figure 1:**
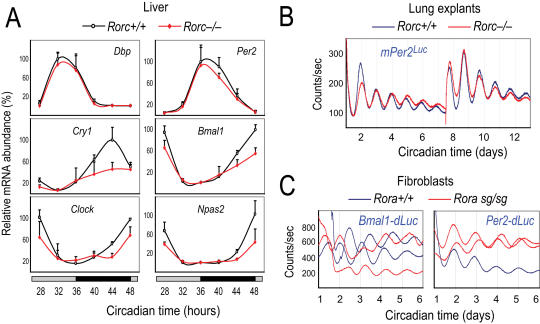
RORc activates Bmal1 transcription but is dispensable for Bmal1 rhythmicity. (A) Circadian *Bmal1* mRNA expression is blunted in the liver of *Rorc* ^−/−^ mice. The peak transcript levels of *Bmal1*, *Clock*, *Npas2*, and *Cry1* are reduced in the liver of Rorc^−/−^ mice compared to WT littermates, suggesting that RORc is an activator of *Bmal1* transcription. Temporal patterns of *Per2* and *Dbp* are unaltered. Expression was analyzed at 4-hr intervals by Q-PCR. Values are expressed as percentage of maximum expression for each gene. Error bars represent standard deviation (SD) of expression levels from four mice. Circadian time: hours after animal release in constant darkness. (B) Representative records of tissue-autonomous *mPer2^Luc^* bioluminescence rhythms in *Rorc* ^−/−^ lung explants. *Rorc* ^−/−^ lung explants displayed normal *mPer2^Luc^* rhythms, suggesting that *Rorc* is not required for circadian rhythmicity. Tissue explants were dissected and immediately cultured in explant medium for recording. Another medium change occurred at day 7. Circadian time: days after explant medium change. (C,D) Representative records of cell-autonomous bioluminescence rhythms in populations of *Rora^sg/sg^* fibroblasts transduced with a lentiviral *Bmal1-dLuc* reporter (C) or *Per2-dLuc* reporter (D). *Rora^sg/sg^* fibroblasts, in which no functional RORa, RORb, or RORc are expressed, displayed rhythmic oscillations of *Bmal1-dLuc* and *Per2-dLuc* reporters. Circadian time: days after explant medium change.

Based on the ability of RORc to activate a *Bmal1-Luc* reporter in vitro and its strong rhythmic expression in many peripheral tissues including the liver and lung [6],[9],[25], we hypothesized that RORc, like RORa and RORb in the SCN, may play an important role as an activator of *Bmal1* in peripheral oscillators. We tested this hypothesis by analyzing *Bmal1* expression in the mouse liver. In *Bcl-xL^Tg^* control mice, *Bmal1* expression peaked at CT24 (Figure 1A). In contrast, *Bmal1* expression at CT28, CT44 and CT48 in the liver of *Rorc^−/−^:Bcl-xL^Tg^* mice was significantly reduced, compared to those of *Bcl-xL^Tg^* siblings (Figure 1A). These results suggest that RORc activates *Bmal1* transcription in the positive arm of the ROR/REV/*Bmal1* loop, functioning to maintain normal amplitude of *Bmal1* rhythmicity.

Although *Bmal1* peak expression levels are reduced in the absence of RORc, *Bmal1* mRNA still retains a rhythm with fairly high amplitude, indicative of functional redundancy from RORa and/or contributions from the REV-ERBs. RORc also regulates transcription of *Cry1*, *Clock* and *Npas2*, all of which are considered RORE-containing genes [30],[31], and their mRNAs were also reduced during peaking hours (Figure 1A). Despite the blunted rhythm amplitudes for *Bmal1*, *Clock*, *Npas2* and *Cry1*, cyclic expression of *Per2* and *Dbp*, however, was not dramatically affected by *Rorc* deletion, similar to observations in *Rev-erbα^−/−^* mice [5].

### RORc Is Not Required in Peripheral Clock Function

As the SCN clock functions normally in the absence of *Rorc*, we assessed the effect of *Rorc* deletion on peripheral clock function in tissue-autonomous preparations in which confounding influences from the SCN are eliminated. Tissue explants of the lung from *Bcl-xL^Tg^* control mice displayed persistent *mPer2^Luc^* rhythms (τ = 24.00 hr±0.33, n = 4). *Rorc^−/−^:Bcl-xL^Tg^* lung explants exhibited rhythmic *mPer2^Luc^* expression with comparable period lengths to controls (τ = 24.15±0.49, n = 4) (Figure 1B). *Rorc^−/−^:Bcl-xL^Tg^* liver explants also displayed persistent *mPer2^Luc^* rhythms (τ = 22.59 hr±1.54, n = 5), similar to controls (τ = 22.22 hr±0.71, n = 3). Surprisingly, no significant differences in circadian amplitude or damping rate were observed between controls and *Rorc* ^−/−^ mice. The normal bioluminescence rhythms are consistent with unaltered molecular phenotypes of *Per2* expression (Figure 1A). Moreover, we observed normal rhythms in fibroblasts, in which *Rorc* expression is not detectable (data not shown), further confirming results from liver and explants. In fibroblasts, over-expression of *Rorc* did not affect *Bmal1* rhythms (data not shown). These results demonstrate that RORc does not play an essential role in maintaining circadian oscillation and suggest that a high-amplitude *Bmal1* rhythm may not be critically required for basic clock operation, similar to phenotypes observed for *Rev-erbα* deficiency [5].

### The ROR Activators Are Not Required for Bmal1 Rhythmicity in Fibroblasts

So far, data suggest a functional redundancy among RORa, RORb and RORc. In the liver and fibroblasts of both *Rora^sg/sg^* [6],[11] and *Rorc* ^−/−^ mice, *Bmal1* peak expression is reduced, but the mRNA rhythm is largely retained and *Per2* oscillation is not altered. Although *Rora* does not show strong rhythmicity in the liver, its expression alone could partially complement the loss of *Rorc*. To study the ROR redundancy genetically, a mouse line deficient in both *Rora* and *Rorc* would represent an ideal reagent. However, such a line is extremely difficult to obtain because *Rora^sg/sg^* mutant mice display cerebellar ataxia and mostly infertile [18] and *Rorc* ^−/−^ mice also have strongly abnormal phenotypes [29]. Therefore, we decided to address the ROR redundancy using *Rora^sg/sg^* fibroblasts. Because *Rorb* and *Rorc* are also not expressed in *Rora^sg/sg^* fibroblasts as determined by Q-PCR (data not shown), thus excluding the possibility of a compensation mechanism, the positive arm of the ROR/REV/*Bmal1* loop is essentially missing in cells lacking *Rora* function.

To monitor the function of the core loop and the ROR/REV/*Bmal1* loop in parallel, we generated two lentivirus-mediated circadian reporters, *pLV6-Per2-dLuc* and *pLV6-Bmal1-dLuc*, designed to report the E-box and RORE-regulated rhythms, respectively. As expected, WT cells displayed persistent *Bmal1-dLuc* rhythms (τ = 24.44±1.55 hr, n = 17 culture dishes from 2 independent cell lines). Importantly, *Rora^sg/sg^* fibroblasts also displayed rhythmic *Bmal1-dLuc* oscillations (τ = 24.34±0.95 hr, n = 30 from 3 lines), comparable to WT cells (Figure 1C). Not surprisingly, these cells also exhibited *Per2-dLuc* rhythms similar to those of WT cells (Figure 1C). Our results demonstrate that the ROR activators contribute to *Bmal1* rhythm amplitude, but are clearly not required for *Bmal1* rhythmicity and core clock function in fibroblasts.

### REV-ERBα and β Are Required for Bmal1 Rhythmicity in Fibroblasts

**Figure 2:**
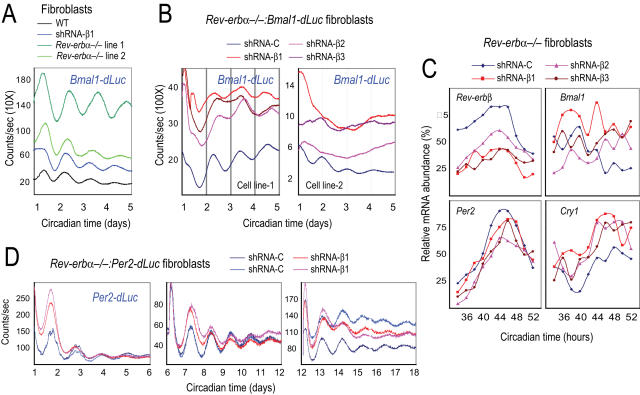
REV-ERBα and β are required for Bmal1 rhythms in fibroblasts. (A) Representative bioluminescence rhythms from a *Bmal1-dLuc* reporter in fibroblasts deficient in either *Rev-erbα* or *Rev-erbβ* function. We tested two independent *Rev-erbα^−/−^* fibroblast cell lines and cells stably expressing an shRNA construct against *Rev-erbβ*. Fibroblasts deficient in either *Rev-erbα* or *Rev-erbβ* function alone displayed rhythmic oscillations of *Bmal1-dLuc* bioluminescence, suggesting functional redundancy of *Rev-erbα* and *Rev-erbβ*. Circadian time: days after explant medium change. (B) Representative bioluminescence patterns from a *Bmal1-dLuc* reporter in fibroblasts deficient in both *Rev-erbα* and *Rev-erbβ* function. *Rev-erbα^−/−^* fibroblasts stably expressing a non-specific control shRNA (shRNA-C) displayed circadian *Bmal1-dLuc* rhythms, but *Rev-erbα^−/−^:Rev-erbβ-*shRNA cells were arrhythmic, suggesting that the REV-ERBs are required for *Bmal1* rhythmic expression. Three different shRNA constructs (shRNA-β1, β2 and β3) were used for knocking down endogenous *Rev-erbβ* expression in fibroblasts. Circadian time: days after explant medium change. (C) Temporal mRNA expression profiles of clock genes in *Rev-erbα^−/−^* fibroblasts stably expressing shRNA constructs against *Rev-erbβ*. Expression was analyzed at 2-hr intervals by Q-PCR. Values are expressed as percentage of maximum expression for each gene. Results were confirmed in two independent time courses. Similar results were obtained from both cell lines, and results for cell line-2 are presented here. For clarity, error bars representing SD of two culture samples for each cell line (<10%) were omitted. *Rev-erbβ* mRNA was significantly reduced by shRNA against *Rev-erbβ*, leading to higher expression levels of *Bmal1* and *Cry1*. *Per2* mRNA rhythms were unaltered in cells deficient in *Rev-erbα* and *β* function. Circadian time: hours after serum treatment. (D) Representative bioluminescence rhythms from a *Per2-dLuc* reporter in fibroblasts deficient in both *Rev-erbα* and *Rev-erbβ* function. *Rev-erbα^−/−^* fibroblasts expressing shRNA constructs against *Rev-erbβ* displayed *Per2-dLuc* rhythms similar to those of shRNA control cells. Similar results were obtained from all three shRNA constructs in two *Rev-erbα^−/−^* fibroblast cell lines, and results from cell line-2 are presented here. The three panels show patterns for the same cultures after three successive medium changes. Circadian time: days after explant medium change.

Next, we examined the consequence of disrupting the negative arm of the ROR/REV/*Bmal1* loop. *Bmal1* expression is significantly higher in the liver [5] and fibroblasts of *Rev-erbα^−/−^* mice than in WT (data not shown). Given the abnormal *Bmal1* expression in the liver and fibroblasts, we expected that deletion of *Rev-erbα* would dramatically compromise the *Bmal1* rhythm, as previously suggested from mRNA analysis [5]. Surprisingly, however, *Rev-erbα^−/−^* fibroblasts displayed rhythmic *Bmal1-dLuc* expression (Figure 2A). The period lengths for *Rev-erbα^−/−^* fibroblasts harboring *Per2-dLuc* reporter were determined to be 26.59±0.29 hr (n = 10) for cell line-1 and 24.25±0.72 hr (n = 10) for cell line-2, and the corresponding WT fibroblasts exhibited a periodicity of 24.88±1.48 hr (n = 7). Thus, as expected, real-time longitudinal bioluminescence recording reveals the dynamics of gene expression, while mRNA profiling lacks temporal resolution and is generally more subject to noise. Given the apparent redundant contribution from *Rev-erbβ*, *Bmal1* rhythms in the liver and lung of *Rev-erbα^−/−^* mice are also likely to be rhythmic, similar to that observed in fibroblasts.

We assessed any redundant contribution from *Rev-erbβ* using small hairpin RNAs (shRNA). We designed and tested nine shRNA constructs against different regions of the *Rev-erbβ* gene, and three of them (shRNA-β1, β2 and β3) were found to be functional in efficiently knocking down *Rev-erbβ* expression (Figure 2C). We introduced *Rev-erbβ*-shRNA constructs into WT fibroblasts harboring *Bmal1-dLuc* reporter. Knockdown of *Rev-erbβ* resulted in higher *Bmal1* mRNA expression, with shRNA-β1 being the most potent (Figure 2C); these cells displayed rhythmic *Bmal1-dLuc* expression (Figure 2A), similar to effects of *Rev-erbα*-knockout. Thus, *Rev-erbα* and *Rev-erbβ* are functionally redundant and disruption of either one alone is not sufficient to disrupt *Bmal1* rhythms.

To disrupt the function of REV-ERBα and β simultaneously, *Rev-erbβ-*shRNA constructs were stably introduced into *Rev-erbα^−/−^* fibroblasts harboring the *Bmal1-dLuc* reporter to obtain *Rev-erbα^−/−^:Rev-erbβ-*shRNA:*Bmal1-dLuc* cell lines. In striking contrast to rhythmic *Bmal1-dLuc* expression in *Rev-erbα*-knockout or *Rev-erbβ*-knockdown fibroblasts, cells deficient in both *Rev-erbα* and *β* function displayed significantly higher levels but largely arrhythmic *Bmal1-dLuc* expression (Figure 2B). For cell line-2, 15/18 dishes of *Rev-erbα^−/−^* cells expressing control shRNA displayed rhythmic *Bmal1-dLuc* expression (FFT spectral amplitude = 0.80±0.08, n = 15), but only 6/19 of *Rev-erbα^−/−^:Rev-erbβ-*shRNA-β1 showed any rhythms, and those that were rhythmic showed significantly lower spectral amplitude (FFT spectral amplitude = 0.50±0.08, n = 6). The weak rhythms may likely result from residual levels of REV-ERBβ expression in these knockdown cells. Similar results were observed in cell line-1 (data not shown). These results demonstrate that the REV-ERBα and β are required for rhythmic *Bmal1* expression in fibroblasts. The finding that cells lacking ROR function retain *Bmal1-dLuc* rhythms whereas those deficient in REV-ERB function are arrhythmic, suggests that the REV-ERB repressors play more prominent roles than the ROR activators in the ROR/REV/*Bmal1* loop.

### *Rev-erbα* and β Are Not Required for PER/CRY Core Loop Function

Given that the *Bmal1-dLuc* reporter is rhythmic in *Rev-erbα^−/−^* fibroblasts, it is not surprising to observe that the *Per2-dLuc* reporter was also rhythmic (Figure 2D). However, it was not known whether disrupting both *Rev-erbα* and *β* would affect the core feedback loop function. We thus introduced *Rev-erbβ-*shRNA constructs into *Rev-erbα^−/−^:Per2-dLuc* fibroblasts and demonstrated that *Rev-erbα^−/−^:Rev-erbβ-*shRNA cells also displayed rhythmic patterns of *Per2-dLuc* expression (τ = 25.99±0.40 hr, n = 7 for cell line-1; τ = 25.12±0.60 hr, n = 22 for cell line-2), similar to cells expressing control shRNA (τ = 26.48±0.27 hr, n = 7 for cell line-1; τ = 25.31±0.52 hr, n = 23 for cell line-2) (Figure 2D).

We also examined effects of *Rev-erbβ*-knockdown on the expression of other clock genes. In shRNA control cells, peaks of *Rev-erbβ* and *Per2* mRNAs (CT40–48) were almost anti-phasic to *Bmal1* (CT32–36). *Bmal1* mRNA was effectively de-repressed, especially at CT46–52 when *Bmal1* was at its nadir in control cells (Figure 2C). Consistent with rhythmic *Per2-dLuc* bioluminescence expression, the *Per2* mRNA expression pattern was essentially the same in *Rev-erbα^−/−^* cells expressing control shRNA and in those expressing shRNA against *Rev-erbβ*.

Given that *Cry1* is under combinatorial regulation by both BMAL1/CLOCK and REV-ERBs [5],[30],[31], we expected that disruption of REV-ERB function would alter the *Cry1* expression pattern. Indeed, compared to WT cells, *Cry1* mRNA levels were higher in *Rev-erbα^−/−^* fibroblasts (data not shown), and even higher in *Rev-erbα^−/−^:Rev-erbβ-*shRNA fibroblasts (Figure 2C), all consistent with REV-ERB proteins being repressors. Although interference with the REV-ERBs clearly disrupted the *Bmal1* rhythm, it did not seem to substantially alter the rhythm of *Cry1* mRNA. *Cry1* mRNA remained to be rhythmic, reaching its nadir at CT36–40 and peaking at CT46–50, illustrating the resilience of the intracellular clock mechanism. It is possible that, even though the *Bmal1* rhythm is abolished, the residual level of REV-ERBβ in the cells was sufficient for combinatorial regulation of *Cry1*. It is also possible that other unknown mechanisms contribute to *Cry1* regulation. This ambiguity can be resolved in future studies by examining cells completely deficient in both *Rev-erbα* and *β* function. Nevertheless, our results suggest that REV-ERBα and β are required for rhythmic expression of *Bmal1*, but REV-ERB function and the *Bmal1* rhythm are not required for normal oscillations of *Per* and *Cry*.

### Constitutive BMAL1 Restores Circadian Rhythmicity in Bmal1^−/−^ Fibroblasts

**Figure 3:**
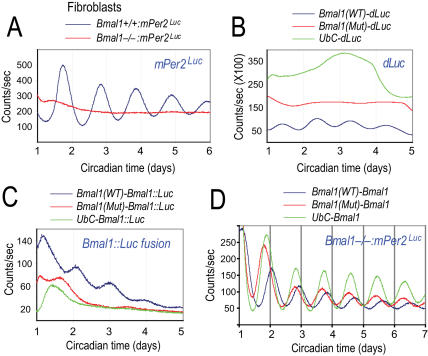
Cyclic expression of BMAL1 is not required for intracellular core clock function. (A) Bioluminescence patterns of fibroblasts derived from WT and *Bmal1^−/−^:mPer2^Luc^ mice*. *Bmal1^−/−^* fibroblasts are completely arrhythmic, suggesting that *Bmal1* is required for clock function in fibroblasts. Circadian time: days after explant medium change. (B) Bioluminescence patterns in wild-type fibroblasts transduced with a lentiviral *dLuc* reporter. *Bmal1*(WT): *Bmal1* promoter containing WT RORE sequence. *Bmal1*(Mut): *Bmal1* promoter containing mutated RORE sequences. *UbC*: Ubiquitin C promoter. Unlike the *Bmal1*(WT), the *Bmal1*(Mut) and *UbC* promoters do not confer rhythmic luciferase expression. Circadian time: days after explant medium change. (C) Bioluminescence patterns in wild-type fibroblasts transduced with a lentiviral *Bmal1::Luc* fusion reporter. Unlike the *Bmal1*(WT), the *Bmal1*(Mut) and *UbC* promoters do not confer rhythmic BMAL1::LUC fusion protein expression. These results suggest that BMAL1 protein does not cycle and that only promoters that contain functional circadian elements confer rhythmic fusion protein expression. Circadian time: days after explant medium change. (D) Representative records of *mPer2^Luc^* rhythms in *Bmal1^−/−^* fibroblasts restored through genetic complementation. Lentiviral expression vectors carrying *Bmal1* cDNA under control of different promoters were introduced into *Bmal1^−/−^:mPer2^Luc^* fibroblasts. The three promoters gave rise to similar levels of BMAL1 protein expression as determined by Q-PCR and Western blotting (data not shown). Both cyclically and constitutively expressed BMAL1 restored circadian rhythmicity in *Bmal1^−/−^* fibroblasts, suggesting that the rhythm of BMAL1 protein is not required for basic core clock function. Circadian time: days after explant medium change.

To further test the role of RORE-mediated *Bmal1* regulation, we eliminated all influences of the RORs and REV-ERBs on *Bmal1* expression in cell-based genetic complementation experiments. Fibroblasts derived from *Bmal1^−/−^:mPer2^Luc^* mice displayed arrhythmic patterns of bioluminescence expression, demonstrating that *Bmal1* is an essential clock component for cellular rhythmicity in fibroblasts (Figure 3A). We asked whether constitutively expressed BMAL1 in *Bmal1^−/−^* fibroblasts could restore circadian rhythmicity. This approach precludes residual REV-ERBβ function from shRNA knockdown and circumvents any off-target effects.

To manipulate *Bmal1* expression, we used three promoters: *Bmal1*(WT) contains a 526-bp DNA fragment from the *Bmal1* promoter encompassing ROREs, *Bmal1*(Mut) is identical to *Bmal1*(WT) except that the RORE sites are mutated to prevent ROR/REV-ERB from binding, and *UbC* is a commonly used constitutive promoter from the *UbC* gene. We showed that WT fibroblasts transduced with a lentiviral *Bmal1*(WT)-*dLuc* reporter displayed rhythmic bioluminescence expression, but *Bmal1*(Mut) or *UbC* promoters did not confer rhythmicity in these cells (Figure 3B).

We next determined the ability of the promoters to regulate the expression of *Bmal1*. In lieu of Western blot analysis of BMAL1, we monitored the bioluminescence expression of BMAL1::LUC fusion protein. We demonstrated that BMAL1::LUC cycled only when it is driven by *Bmal1*(WT), and that *UbC* and *Bmal1*(Mut) promoters did not confer rhythmic fusion protein expression (Figure 3C). Thus, BMAL1 protein itself does not cycle in the absence of a RORE-containing circadian promoter.

To carry out genetic complementation, we generated a lentiviral expression vector *Bmal1*(WT)-*Bmal1*·Flag, in which *Bmal1* cDNA is under the control of WT *Bmal1* promoter. When this construct was introduced into *Bmal1^−/−^:mPer2^luc^* fibroblasts, circadian rhythmicity was restored (τ = 22.02±0.68 hr, n = 25 cultured dishes) (Figure 3D), but not in cells expressing a *Bmal1*(WT)-*GFP* control construct (data not shown). Importantly, non-cyclically expressed BMAL1 under the control of either *UbC* or *Bmal1*(Mut) also effectively restored circadian *mPer2^Luc^* rhythmicity in *Bmal1^−/−^* fibroblasts (τ = 22.08±0.46 hr, n = 20 for *UbC-Bmal1*; τ = 22.61±0.60 hr, n = 27 for *Bmal1*(Mut)-*Bmal1*) (Figure 3D). Taken together, these results demonstrate that rhythmic expression of BMAL1 protein is not essential for the basic functioning of the intracellular clock. These results provide the cellular basis for the finding that constitutive *Bmal1* expression was able to rescue circadian behavioral rhythms in *Bmal1^−/−^* mice [24].

### In Vivo Genetic Evidence for Cry1 and Rorc Regulation by REV-ERBs

**Figure 4:**
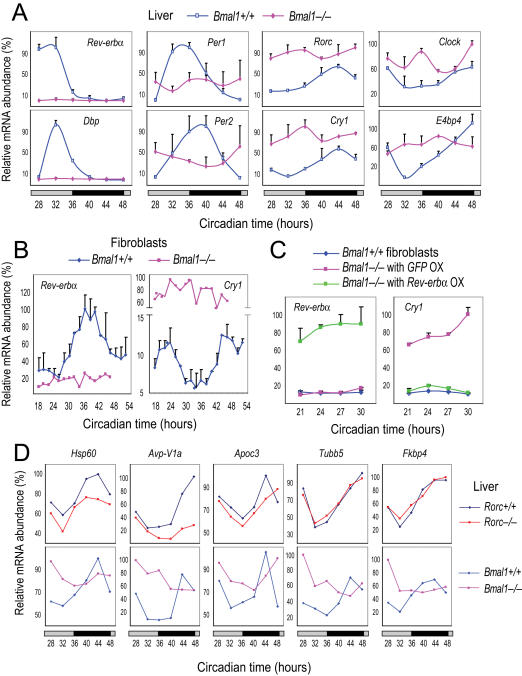
REV-ERBs play a prominent role in combinatorial regulation of Cry1 and Rorc. (A) Temporal mRNA expression profiles of clock genes in the liver of *Bmal1^−/−^* mice. Expression was analyzed at 4-hr intervals by Q-PCR. Values are expressed as percentage of maximum expression for each gene. Error bar represents standard deviation (SD) of expression levels from four mice. The clock genes are presented in four groups based on different mRNA expression patterns (phase and level) in WT and *Bmal1^−/−^* mice. For instance, transcription of *Cry1* and *Rorc* is elevated, rather than repressed, in the *Bmal1^−/−^* liver. Circadian time: hours after animal release in constant darkness. (B) Temporal mRNA expression profiles of *Rev-erbα* and *Cry1* in *Bmal1^−/−^* fibroblasts. Expression was analyzed at 2-hr intervals by Q-PCR. Values are expressed as percentage of maximum expression for each gene. Results were confirmed in two independent time courses. Error bars represent SD of two culture samples for each cell line. *Cry1* mRNA levels are constantly high throughout the day and *Rev-erbα* expression is completely abolished in *Bmal1^−/−^* fibroblasts, similar to results obtained from the liver. Circadian time: hours after serum treatment. (C) Over-expression (OX) of *Rev-erbα* represses elevated *Cry1* mRNA levels in *Bmal1^−/−^* fibroblasts. Expression of GFP and REV-ERBα is driven by a constitutive *CAG* promoter. Temporal mRNA expression was analyzed at 3-hr intervals by Q-PCR. Values are expressed as percentage of maximum expression for each gene. Results were confirmed in two independent experiments. Error bars represent SD of two culture samples for each cell line. REV-ERBα expression was confirmed by Q-PCR, and also by Western blotting (data not shown). Circadian time: hours after serum treatment. (D) Temporal mRNA expression profiles of clock-controlled output genes in the liver of *Rorc* ^−/−^ and *Bmal1^−/−^* mice. Experiments were performed as described in Figure 1A for *Rorc* ^−/−^ mice and Figure 4A for *Bmal1^−/−^* mice. As for *Bmal1* and *Cry1*, the prominent role of REV-ERBs in regulating transcription explains the elevated mRNA levels of these output genes in *Bmal1^−/−^* mice. For clarity, error bars representing SD from four mice (<10% for each gene) were omitted. Circadian time: hours after animal release in constant darkness.

The *Rorc* gene has at least two E-boxes within the promoter region, and its circadian expression pattern is similar to *Cry1* in the liver. In vitro studies suggest that *Rorc* transcription is regulated by BMAL1/CLOCK [31]. To verify the in vitro results, we demonstrated that, similar to the expression patterns of other BMAL1/CLOCK-regulated clock components, the *Rorc* mRNA rhythm was abolished in the *Bmal1^−/−^* mouse liver, confirming that *Rorc* is regulated by the core loop (Figure 4A).

Interestingly, however, we observed that mRNA levels of *Rorc* as well as *Cry1* are clearly elevated rather than reduced in *Bmal1^−/−^* liver. This was surprising at first given that BMAL1 is a known activator of *Cry1* and *Rorc* expression. However, it should not be so surprising given the complexity of transcriptional circuitry of the clock. Similarly, higher *Cry1* mRNA levels were also reported previously in *Bmal1^−/−^*, *Clock^m/m^* and *Clock^−/−^* mice [32],[33]. A recent in silico study showed that *Cry1* and *Rorc* genes contain two types of circadian regulatory elements, the E-box and the RORE [31]. In vitro and in vivo evidence also supports the presence of RORE sites within the *Cry1* gene [5],[30]. In the absence of E-box regulation, factors acting through the RORE, namely the RORs and REV-ERBs, are likely to govern *Cry1* and *Rorc* transcription. In line with this notion, *Clock* mRNA is also higher in *Bmal1^−/−^* liver (Figure 4A), and *Bmal1* mRNA is higher in *Clock^−/−^* mouse liver [33].

**Figure 5:**
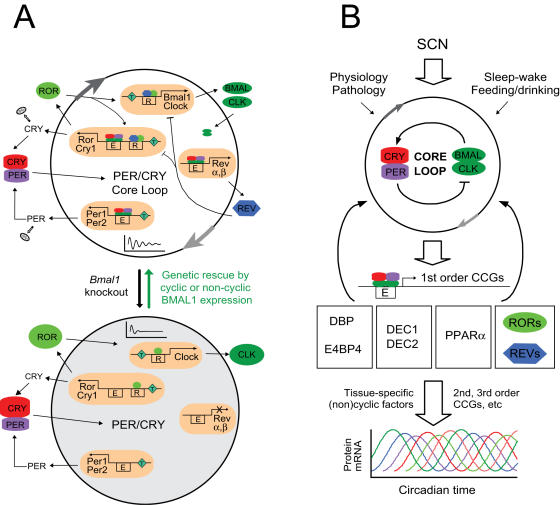
Model for circadian core clock mechanism and function. (A) Different transcriptional regulation gives rise to differential phasing of clock genes. PER/CRY and BMAL1/CLOCK (BMAL/CLK) form the core feedback loop mediated by the E-box. The RORs and REV-ERBs are directly regulated by the core loop and provide additional positive and negative feedbacks, respectively, to *Bmal1*/*Clock* transcription. Four main types of gene regulatory mechanisms exist in a wild-type cell (top): 1) *Rev-erbα* and *β* (Rev) are driven primarily by E-box-mediated transcription, 2) *Per1* and *Per2* are regulated by BMAL1/CLOCK and additionally by a tonic signal input (T), 3) *Cry1* and *Rorc* are regulated by BMAL1/CLOCK and ROR/REV as well as a tonic signal, and 4) *Bmal1* and *Clock* are regulated by ROR/REV and a tonic signal. These different modes of transcriptional regulation provide the mechanistic basis for the different phases of their mRNAs (e.g. *Rorc* phase-delays *Rev-erbα*) in WT cells and the differential levels of expression (e.g., diminished REV leads to *Cry1* up-regulation) in *Bmal1^−/−^* cells. BMAL1 is an essential clock component, and *Bmal1^−/−^* cells are completely arrhythmic (bottom). However, its rhythmic patterns of mRNA and protein expression are not required for core clock function. Genetic complementation by either cyclically or constitutively expressed *Bmal1* was able to restore circadian rhythmicity in *Bmal1^−/−^* cells. We suggest that the robustness of the core loop in the absence of rhythmic BMAL1 is retained by coordinated regulation of transcriptional and post-translational mechanisms, including particularly protein turnover and synchronous nuclear translocation of PER/CRY proteins despite the differential phases and/or lack of rhythmicity of their mRNAs (see discussion). In both the core loop and ROR/REV/*Bmal1* loop, the repressors play more dominant roles than the activators. (B) The interlocking loops connect the core loop to temporal regulation of local output networks. Peripheral tissues are coordinated by the SCN in vivo, and the states of peripheral oscillators are also influenced by behavior, physiology, and pathology. The core loop directly controls expression of 1^st^ order CCGs, subsequently forming a cascade of rhythmic gene expression. The net result of this cascade is the appropriately timed production of proteins important for local physiology, which collectively contribute to coordinated circadian behavior and physiology at the organismal level. In this context, the interlocking loops, including the ROR/REV/*Bmal1* loop and its constituents, are 1^st^ order CCGs and serve as important transmitters or integrators for local circadian biology.

A recent study proposed dual activator and repressor functions of BMAL1/CLOCK, in which its repressor function explains the elevated *Cry1* expression in the absence of *Bmal1* [32]. However, that study did not take into consideration *Cry1* gene regulation through the ROREs. In both WT and *Bmal1^−/−^* mouse liver, there exists a strong inverse correlation between *Rev-erbα* and *Cry1*/*Rorc* mRNA levels: when *Rev-erbα* is high, *Cry1*/*Rorc* is low, and vice versa (Figure 4A). Similar expression patterns were also observed in fibroblasts (Figures 1B and 5B) and in *Rev-erbα^−/−^* mice [5], and suggested from in silico and in intro studies [30],[31]. Thus, the elevated *Rorc* and *Cry1* expression in the absence of *Bmal1* may be regulated primarily by the REV-ERBs rather than the repressor function of BMAL1. We therefore sought to experimentally demonstrate this notion. We hypothesized that over-expression of *Rev-erbα* in *Bmal1^−/−^* cells would bring down the expression levels of *Cry1* and *Rorc*. Because *Cry1* and *Rorc* genes are regulated similarly but *Rorc* is not expressed in fibroblasts, we focused our analysis on the *Cry1* gene in this cell type. To test this idea, we introduced *Rev-erbα* into *Bmal1^−/−^* cells by lentivirus-mediated delivery and obtained a *Bmal1^−/−^:Rev-erbα*-OX fibroblast cell line. Indeed, over-expressed REV-ERBα in *Bmal1^−/−^* cells efficiently repressed *Cry1* mRNA to levels similar to those in WT cells (Figure 4C). Taken together, we provide direct in vivo genetic and molecular evidence to support the notion that *Cry1* and *Rorc* are regulated not only by BMAL1/CLOCK but also directly by the REV-ERBs (Figure 5A), which is the underlying molecular mechanism for elevated *Cry1* expression in *Bmal1^−/−^* cells.

### Different Transcriptional Regulation Explains Differential Phasing of Clock Genes

Interestingly, the mRNA levels of other clock genes in the liver of *Bmal1^−/−^* mice are also very different (Figure 4A): *Dbp* and *Rev-erbα* expression is dramatically reduced, and *Per1* and *Per2* are expressed at constant intermediate levels, consistent with sustained *mPer2^Luc^* expression in *Bmal1^−/−^* cells (Figure 3A), whereas *Rorc*, *Cry1*, *Clock* and *E4bp4* are clearly de-repressed. Based on mRNA expression patterns in both WT and *Bmal1^−/−^* cells (Figure 4A), we suggest the following transcriptional regulatory scheme for clock gene expression (Figure 5A): *Dbp* and *Rev-erbα* are activated primarily by BMAL1/CLOCK via the E-boxes, and that this E-box-mediated circadian regulation is essentially eliminated in the absence of BMAL1 (and thus PER/CRY-mediated repression via the E-box is no longer relevant). *Per1* and *Per2* are activated by BMAL1/CLOCK and other non-circadian mechanisms, accounting for the intermediate mRNA levels of *Per1* and *Per2* in *Bmal1^−/−^* mice. *Rorc* and *Cry1* are regulated not only by BMAL1/CLOCK but also by RORs/REV-ERBs via the RORE. *Bmal1*, *Clock* and *E4bp4* are regulated by RORs/REV-ERBs via the RORE.

The different regulatory mechanisms offer mechanistic explanations for distinct phases of clock gene expression rhythms observed in vivo (Figure 5A). *Dbp* is controlled by BMAL1/CLOCK via the E-box, while *E4bp4* is primarily regulated via RORE, explaining why the *E4bp4* rhythm is in phase with *Bmal1* and *Clock*, but is antiphasic to *Dbp*. *Rev-erbα* and *Rorc* are both activated by BMAL1/CLOCK, but *Rorc* is also repressed by REV-ERBs, explaining how *Rorc* mRNA accumulation is phase-delayed compared to that of *Rev-erbα*. Additional regulation of *Per1* and *Per2* by non-circadian factors (and possibly also by *E4bp4*) may cause a phase-delay compared to *Dbp* and *Rev-erbα*. In summary, our data provides novel mechanistic insights into how the genes in the clock circuitry are regulated in vivo [31].

### RORc and REV-ERBs Control Rhythmic Expression of Output Genes

The RORs appear to regulate the amplitude of target gene expression, while the REV-ERBs regulate the rhythmic expression of *Bmal1* and also participate in combinatorial regulation of *Cry1*. As these regulatory mechanisms are not required for basic clock function, we suggest that the ROR/REV/*Bmal1* loop and its constituents provide additional opportunities to control time-specific expression of output genes in local clock physiology, especially in peripheral tissues. In this context, the differential tissue expression patterns of the RORs also provide additional opportunities for tissue-specific local circadian biology (Figure 5B).

The combinatorial regulatory mechanism provides a novel strategy for identifying and validating target genes of the RORs and REV-ERBs, as well as differentiating RORE-containing genes from those containing both RORE and E-boxes (Figure 4A). Here we examined several of the genes that exhibit phases similar to *Bmal1* or *Cry1* in the liver and contain potential RORE sequences [25],[26]. For example, mRNAs of heat-shock protein 60 (*Hsp60*), arginine vasopressin receptor 1A (*Avp-V1a*) and *Apoc3* were reduced in the liver of *Rorc* ^−/−^ mice, especially at peak time (CT40–48), reflecting reduction of RORE-mediated activation, but their mRNA levels were up-regulated in *Bmal1^−/−^* mice at CT28–36, corresponding to the trough time in WT, reflecting loss of E-box-mediated REV-ERB expression with subsequent relief of RORE-mediated repression. Tubulin beta 5 (*Tubb5*) and peptidyl-prolyl cis-trans isomerase FK506 binding protein 4 (*Fkbp4*) also exhibited significantly higher mRNA levels at CT28–36 in *Bmal1^−/−^* mice, but their expression levels were not affected in *Rorc* ^−/−^ mice. Thus, cyclic RORE-mediated activation and/or repression may modulate expression patterns of specific target genes involved in important biological processes in a tissue-specific manner.

## Discussion

In summary, our results suggest that the intracellular core clock loop is intrinsically resilient and is largely responsible for generating and maintaining basic circadian rhythmicity. The multiple additional interlocking loops contribute to, but are not necessary for, core clock function. Cellular rhythms are intrinsically stochastic. However, intercellular coupling mechanisms uniquely present in the SCN play a dominant role in maintaining the robustness of the SCN and the body timekeeping system [2],[23]. We therefore suggest that interlocking loops function mainly to provide additional regulatory mechanisms to modulate the phases of gene expression locally.

### Regulation of the Bmal1 Interlocking Loop

Previous studies using mice deficient in *Rora*, *Rorb* or *Rev-erbα* function strongly suggested functional redundancy among the ROR and REV-ERB family members [5],[6],[10],[11]. Mutation of *Rora* was shown to reduce *Bmal1* mRNA amplitude both in the SCN [6] and in fibroblasts [11], and *Rev-erbα* deletion resulted in much higher levels of *Bmal1* transcription [5], but *Bmal1* rhythms were still retained despite either deficiency.

While null mutations in core clock genes typically lead to severe impairment of clock function (see below), deficiencies in clock components within the ROR/REV/*Bmal1* loop only produce modest clock phenotypes [5]–[7],[10],[11]. The ROR/REV/*Bmal1* loop is thus thought to provide a “stabilizing” function. However, mice deficient in core clock components (e.g., *Per1^−/−^*, *Per2^−/−^* or *Clock^m/m^* mice) also similarly show less precise or less persistent circadian rhythms [19]–[22].

In this study, we investigated the redundancy of functions among the ROR and REV-ERB family members and clarified their roles in regulating *Bmal1* expression. To circumvent pleiotropic effects of gene deletion, we directly tested this “stabilization function” hypothesis in cell-autonomous clock models by perturbing the BMAL1 rhythm. We demonstrated that cells with *Rev-erbα*-knockout or *Rev-erbβ*-knockdown still rhythmically express *Bmal1*. The *Bmal1-dLuc* rhythm could be abolished only when both *Rev-erbα* and *β* were disrupted (Figure 2B). Thus, REV-ERBα and REV-ERBβ are required for *Bmal1* rhythmicity, and they are functionally redundant. In contrast, the RORs are not required for *Bmal1* rhythmicity (Figure 1). Thus, the REV-ERBs play a more prominent role than the RORs in regulating the rhythmic expression of *Bmal1*.

### The Robustness of the Circadian Clock

The current models for mouse and fly circadian clocks indicate that the process of evolution has produced a genetic circuitry substantially more complex than a simple transcriptional feedback scheme. Presumably, robustness is a key feature of circadian control that is likely to be under selective pressure, as it would underlie the adaptive significance of a particular physiological rhythm. Robustness is the ability of a system to maintain essential properties despite internal noise and external perturbations, a property which is prevalent in biological control circuits [34]. From a circadian clock perspective, the key measures of robustness are precision (period stability over time), persistence (how long a given clock system sustains rhythm amplitude without a resetting signal), and accuracy (period consistency of cells, tissues, or organisms). It should be noted, however, that period variation and alteration may be an indicator of robustness, not necessarily instability. Mechanisms contributing to the robustness of the clock system include additional interlocking loops, gene redundancy, maintenance of amplitude, and intercellular coupling.

In contrast to the proposed “stabilizing” role of the ROR/REV/*Bmal1* loop, we found that *Per2-dLuc* expression is rhythmic even in cells deficient in both REV-ERBα and β function (Figure 2D) or expressing constitutive BMAL1 protein (Figure 3D). This provides unambiguous evidence from cell-autonomous preparations that *Bmal1* mRNA and protein rhythms are not essential for the basic operation of the intracellular clock. In accord with our findings, constitutively expressed *Bmal1* in the SCN of *Bmal1^−/−^* mice was able to rescue circadian behavioral rhythmicity [24].

Using real-time bioluminescence imaging to monitor *Per2* gene expression in tissues and cells from mutant mice [23], we recently found that both *Per1* and *Per2* are required for sustained cell-autonomous rhythms in individual cells. Importantly, intercellular coupling in the SCN can compensate for clock gene deficiency, preserving sustained cellular rhythmicity in mutant SCN slices and behavior. Thus, SCN intercellular coupling is essential not only to synchronize component cellular oscillators but also for robustness against genetic perturbations. In this context, it is reasonable to presume that, owing to intercellular coupling, an SCN ensemble that expresses non-cyclic *Bmal1* mRNA/proteins would still exhibit robust *Per2* and *Cry1* rhythms. However, *Rora^sg/sg^*, *Rorb^−/−^* and *Rev-erbα^−/−^* mice exhibit circadian period defects in behavior, albeit very mild. Thus, to address the cellular basis of circadian behavior, future studies using real-time bioluminescence technology are needed to examine the molecular dynamics of circadian rhythmicity in the SCN ensemble as well as in dissociated SCN neurons of single and double loss-of-function mutants of the *Ror* and *Rev-erb* genes.

However, the nonessential ROR/REV/*Bmal1* loop in the basic intracellular clock mechanism clearly regulates expression rhythm and amplitude of many output genes. Maintaining a biologically relevant high-amplitude rhythm of gene expression also contributes to the robustness of the clock system. The significance of amplitude in clock function is supported by a recent study showing that *Clock^m/m^* mice exhibited increased efficacy in response to resetting stimuli due to reduced circadian amplitude in the SCN pacemaker [35]. Similarly, the ROR/REV/*Bmal1* loop may also benefit organismal survival in the natural environment by contributing to robust high-amplitude rhythms [13]. Furthermore, this interlocking loop may contribute to transduction of environmental cues to the core loop [13]. In line with this notion, behavioral studies have implicated *Rev-erbα* and *Rorb* in photic responses [5],[36]. It is interesting to note that there appears to be a delayed phase of *Per2* oscillation in cells that express arrhythmic *Bmal1* mRNA and protein (Figures 3D and 4D). As *Per2* induction may be involved in synchronization [37], it is possible that the ROR/REV/*Bmal1* loop plays an important role in circadian entrainment of peripheral oscillators.

### Potential Tonic Signaling Input to Circadian Intracellular Transcriptional Networks

The resilience of the intracellular core clock function without inputs from the ROR/REV/*Bmal1* loop indicates that general cellular mechanisms must play important roles in attaining robust clock function, including particularly post-translational modifications and protein turnover affecting subcellular translocation and activities of clock components. In particular, results from this study strongly suggested the involvement of tonic signaling in clock function (Figure 5A). In *Bmal1^−/−^* cells, transcription of *Rev-erbα* and *β* is completely abolished, whereas *Per1* and *Per2* maintain intermediate transcription levels throughout the day. Any contribution from *Dbp*/*E4bp4* is minimal in these cells, as the level of the DBP activator is too low and the E4BP4 repressor is constantly high (Figure 4A). Rather, it is likely that, without BMAL1/CLOCK activators, *Per1* and *Per2* transcription is maintained through a non-circadian, tonic signal input such as the cyclic AMP response element-binding (CREB) signal transduction cascade. Similarly, presence of tonic signaling and lack of repression by the REV-ERBs are the primary cause for the constantly high levels of *Cry1*, *Rorc* and *Clock* expression in *Bmal1^−/−^* cells. It is conceivable that the activating tonic signal input also explains why the ROR activators are dispensable for driving rhythmic transcription of *Bmal1* provided that the REV-ERBs are present in the cells. It is likely that the balance between positive and negative regulators as well as tonic signaling determines clock gene expression at any given circadian time. The tonic signal input is usually overlooked in the WT genetic background, but is uncovered when the functions of positive and/or negative regulators are blocked (Figure 5A). Tonic signaling is also important to consider in interpreting effects of *Per* or *Cry* mutations on cellular rhythms in the SCN [2],[23].

### Interlocking Loops Function Mainly To Regulate Circadian Outputs

In addition to the ROR/REV/*Bmal1* loop, other known interlocking loops or components include *Dbp*/*E4bp4*, *Pparα*, and *Dec1/Dec2* (Figure 5B). These secondary loops are directly regulated by the core loop through the E-boxes [31]. E4BP4 and DBP, analogs of dVRI and dPDP1 in flies [16], form an oscillatory loop by feeding back to regulate *Per2* transcription [31], [38]–[40]. DEC1 and DEC2 form another feedback loop, functioning to repress E-box-mediated transcription [41]. Very recently, *clockwork orange* (*cwo*), a *Dec* homolog, has been identified in Drosophila and shown to regulate rhythm amplitude [42]–[44]. The PPARα loop, on the other hand, feeds back to activate *Bmal1* expression through potential PPAR response elements in the *Bmal1* gene [45]–[47]. Interestingly, peroxisome proliferator-activated receptor-gamma coactivator 1 alpha (PGC-1α) has recently also been shown to activate *Bmal1* expression by acting as a ROR activator [48].

Unlike the requirement for core clock components—PER/CRY [23], CLOCK [49], and BMAL1 (Figure 3A in this study), none of the interlocking loops discussed above appears to be required for basic clock function (in this study) [47], [48], [50]–[52]. In addition, the ROR/REV/*Bmal1* loop function is not conserved between mammals and flies [7],[16]. Rather, it's conceivable that the major function of the interlocking loops is to transmit circadian signals to control local output genes at different times during the day, as required for circadian behavior and physiology. Direct transduction of circadian information to local output rhythms can be more efficiently accomplished through first-order clock-controlled genes (1^st^ order CCGs) that are directly regulated by the core loop, which subsequently regulate expression of 2^nd^ and 3^rd^ order CCGs (Figure 5B). In this context, the interlocking loops and their constituents serve as the 1^st^ order CCGs. Most CCGs exhibit tissue-specific expression patterns, and many are involved in rate-limiting steps of reactions important for the main functions of their respective tissues [3],[26],[53],[54]. The core loop components are well conserved among various tissues, while the 1^st^ order CCGs such as the RORs may be highly tissue-specific. The 1^st^ order CCGs could also establish crosstalk with other tissue-specific circadian or non-circadian factors. Thus, the multiple interlocking loops provide an efficient means not only to amplify circadian signals but also to provide additional phase information for local outputs (Figure 5B). For instance, the antiphasic expression of *Dbp* and *E4bp4* is known to regulate the rhythmic production of many proteins involved in bile acid production, drug metabolism, and xenobiotic detoxification in liver and kidney [3],[38],[55].

The RORs and REV-ERBs are known to be involved in many cellular, physiological, and pathological processes [4], [56]–[60]. For example, RORa and RORc regulate phase I and II metabolism [61]. RORa activates and REV-ERBα represses genes encoding apolipoprotein C-III (ApoC-III) and ApoA1, key proteins in plasma triglyceride and lipoprotein metabolism [60]. RORa and REV-ERBβ also regulate many genes involved in lipid homeostasis in skeletal muscle cells [62],[63]. REV-ERBα was shown to regulate circadian expression of plasminogen activator inhibitor type 1 (PAI-1), suggesting a role in thrombosis [64]. Interestingly, crosstalk exists between REV-ERBα and PPAR nuclear receptors, which are important factors regulating lipid and glucose homeostasis and inflammation [60]. Recently, anatomical expression profiling of nuclear receptors has revealed significant metabolic implications of peripheral clock biology [27],[28],[65].

With the interlocking loops as entry points, future studies should focus on more detailed characterization of the transcriptional circuitry regulating time- and tissue-specific outputs involved in circadian behavior, physiology, and pathology. Knowledge of circadian signaling and clock-regulated local biology will likely have important implications for the pathogenesis and treatment of diseases such as metabolic syndrome, heart disease, diabetes, and obesity.

## Materials and Methods

### Animal

*Bmal1^−/−^* mouse line was obtained from Chris Bradfield at the University of Wisconsin, *Rorc^−/−^* line from Dan Littman at New York University, and *mPer2^Luc^* transgenic reporter line from Joe Takahashi at Northwestern University. Knockout mice were bred with *mPer2^Luc^* reporter mice to obtain homozygous knockouts harboring the *mPer2^Luc^* reporter. Wheel-running assays were performed and analyzed as described previously [23]. Behavioral phenotypes of these mice were similar to the respective knockout animals not carrying the reporter. All animal studies were conducted in accordance with the regulations of the Committees on Animal Care and Use at The Scripps Research Institute.

### Cell Culture, Transfection, and Lentiviral Production and Infection

Explants of SCN and peripheral tissues were dissected and cultured as previously described [23],[66]. Primary mouse fibroblasts were generated from tails by a standard enzymatic digestion procedure [67]. Fibroblasts that spontaneously overcame replicative senescence (immortalization) were used in this study. All fibroblasts were cultured in DMEM supplemented with 10% fetal bovine serum and antibiotics, and grown to confluence prior to bioluminescence recording or harvesting for mRNA time courses. MMH-D3 hepatocytes were cultured as described previously [68].

Recombinant lentiviruses were produced by transient transfection in 293T cells using the calcium-phosphate method as previously described [23]. Infectious lentiviruses were harvested at 48 hr post-transfection and used to infect various cells. Cells infected with pLL3.7(GW)-shRNA constructs were sorted by FACS for the highest (10%) GFP-expressing cells. Cells infected with pLV156-*Per2-dLuc* reporter [23] were sorted by FACS for GFP expression as described therein. Cells infected with pLenti6-B4B2 constructs expressing proteins including *Per2-dLuc* and *Bmal1-dLuc* reporters were selected with 10 µg/ml Blasticidin and further propagated for further study.

### DNA and shRNA Constructs

For cDNA expression constructs, DNA sequences including *GFP*, *Bmal1*, *Rev-erbα*, *Rev-erbβ*, the firefly *Luciferase* gene (*Luc*), the rapidly *degradable Luciferase* gene (*dLuc*), and *Bmal1::Luc*, were first cloned into pENTR/D-TOPO entry vector (Invitrogen). All promoter sequences including *Bmal1*(WT), *Bmal1*(Mut), *UbC*, *Per2,* and the composite *CAG* promoter were first cloned into pENTR-5′-TOPO vector (Invitrogen). The pENTR/D-TOPO-cDNA and pENTR-5′-promoter plasmid DNAs were then recombined with pLenti6/R4R2/V5-DEST destination vector (Invitrogen) in a MultiSite Gateway recombination reaction to generate expression constructs (see Text S1).

For shRNA expression constructs, we first designed and generated nine 29-bp long oligo-nucleotides against different regions of the *Rev-erbβ* gene. Synthetic oligonucleotides were annealed and cloned into pENTR/U6 (Invitrogen) according to manufacture's instruction and subsequently cloned into the pLL3.7GW vector which harbors a CMV-driven GFP gene [69]. Among the tested nine shRNA constructs against *Rev-erbβ*, three (designated β1, β2 and β3) were found that were non-overlapping and efficiently depleted over-expressed REV-ERBβ protein in transfected HEK293T cells as tested by Western blot analysis (data not shown) and knocked down *Rev-erbβ* mRNA expression in fibroblasts as tested by Q-PCR. The parental pLL3.7GW empty vector and a nonspecific shRNA construct were used as controls (see Text S1).

### Tissue Harvest and Quantitative PCR (Q-PCR)

For liver and lung, mice were first entrained to regular light-dark cycles and then released to constant darkness, and peripheral tissue samples were harvested 28 hr later. Total RNAs from liver and lung were first prepared using Trizol reagents (Invitrogen) followed by further purification using RNeasy mini kit (Qiagen). For fibroblasts, cell growth, serum shock were performed as previously described [5]–[7],[10],[23]. Total RNAs from fibroblasts were prepared using RNeasy mini kit.

Total RNAs were transcribed to cDNA using 1^st^ strand SuperScript III reverse transcriptase (Invitrogen). Q-PCR was performed using an iCycler thermal cycler with the MyiQ optical module (BioRad) as described previously [6],[23]. Transcript levels for each gene were normalized to *Gapdh*. Average relative expression ratios for each gene were expressed as a percentage of the maximum ratio at peak expression (see Text S1).

### Bioluminescence Recording and Data Analysis

Bioluminescence patterns were monitored using a LumiCycle luminometer (Actimetrics) as previously described [23]. Briefly, after change to fresh explant medium at ambient temperature, culture dishes containing cells or explants were sealed and placed into the luminometer, which was kept inside a standard tissue culture incubator at 36°C. Bioluminescence from each dish was continuously recorded with a photomultiplier tube (PMT) for ∼70 sec at intervals of 10 minutes. Raw data (counts/sec) were plotted against time (days) in culture. For analysis of rhythm parameters, we used the LumiCycle Analysis program (Actimetrics). Raw data were baseline fitted, and the baseline-subtracted data were fitted to a sine wave (damped), from which the period was determined. For samples that showed persistent rhythms, goodness-of-fit of >80% was usually achieved. Due to high transient luminescence upon medium change, the first cycle was usually excluded from rhythm analysis. For FFT spectral analysis (RelAmp) of *Bmal1-dLuc* oscillations, LumiCycle Analysis version 2.10 was used, in which polynomial order was set at 3 for background subtraction, the first cycle of data was usually excluded, Blackman-Harris windowing was checked (power spectrum unchecked), and circadian range was defined at 20–30 hr.

## Supporting Information

Figure S1 ROR/REV-ERB expression patterns and *Rorc-/-* animal behavioral rhythms. (A) Tissue-specific expression of the *Ror-* and *Rev-erb* genes. Total RNA was isolated from the tissues indicated, and gene expression was determined by standard reverse transcription and PCR (RT-PCR) followed by agarose gel electrophoresis. (B) Temporal mRNA expression profiles of *Bmal1*, *Dbp*, *Rora*, and *Rorc* in wild-type fibroblasts and hepatocytes. Expression was analyzed at 4-hr intervals by quantitative PCR (Q-PCR). Values are expressed as percentage of maximum expression for each gene. Error bars represent standard deviation (SD) of expression levels from two culture samples. Circadian time: hours after serum treatment. (C) Summary of *Rora*, *Rorb*, and *Rorc* expression in the SCN, liver, and fibroblasts. Curved line: rhythmic expression. Flat line: arrhythmic expression. NE: not expressed or expression not detected. Note that in fibroblasts, *Rorb* and *Rorc* are not detected, and *Rora* expression does not display a distinct mRNA rhythm. *Rorc* is not expressed in the SCN, but is rhythmically expressed in liver. (D) Double-plot actograms for *Bcl-xLTg* controls and homozygous *Rorc-/-:Bcl-xLTg* mice. *Rorc-/-* mice displayed normal circadian locomotor activity under constant darkness and normal phase shifts in response to a light pulse, compared to controls. Yellow shading represents the light period of LD cycles. Red arrows indicate a light pulse applied at CT16.
(1.26 MB PDF)

Text S1 Supplemental Materials and Methods. (A) Expression vector construction. (B) shRNA vector construction. (C) TaqMan PCR primers and probes used in this study.
(0.10 MB PDF)
