# Supplementary material for: Redundant Function of REV-ERBα and β and Non-Essential Role for Bmal1 Cycling in Transcriptional Regulation of Intracellular Circadian Rhythms
Source: PLoS Genet. 2008 Feb 29;4(2):e1000023. doi: 10.1371/journal.pgen.1000023 (PMC2265523; doi:10.1371/journal.pgen.1000023)
Supplement: Figure S1 — ROR/REV-ERB expression patterns and Rorc-/- animal behavioral rhythms. (A) Tissue-specific expression of the Ror- and Rev-erb genes. Total RNA was isolated from the tissues indicated, and gene expression was determined by standard reverse transcription and PCR (RT-PCR) followed by agarose gel electrophoresis. (B) Temporal mRNA expression profiles of Bmal1, Dbp, Rora, and Rorc in wild-type fibroblasts and hepatocytes. Expression was analyzed at 4-hr intervals by quantitative PCR (Q-PCR). Values are expressed as percentage of maximum expression for each gene. Error bars represent standard deviation (SD) of expression levels from two culture samples. Circadian time: hours after serum treatment. (C) Summary of Rora, Rorb, and Rorc expression in the SCN, liver, and fibroblasts. Curved line: rhythmic expression. Flat line: arrhythmic expression. NE: not expressed or expression not detected. Note that in fibroblasts, Rorb and Rorc are not detected, and Rora expression does not display a distinct mRNA rhythm. Rorc is not expressed in the SCN, but is rhythmically expressed in liver. (D) Double-plot actograms for Bcl-xLTg controls and homozygous Rorc-/-:Bcl-xLTg mice. Rorc-/- mice displayed normal circadian locomotor activity under constant darkness and normal phase shifts in response to a light pulse, compared to controls. Yellow shading represents the light period of LD cycles. Red arrows indicate a light pulse applied at CT16. (1.26 MB PDF) [file pgen.1000023.s001.pdf]

Liu AC, et al., *PLoS Genetics*, 2008

"Redundant Function of REV-ERBα and β and Non-Essential Role for Bmal1 Cycling in Transcriptional Regulation of Intracellular Circadian Rhythms"

Supporting Information

Supplemental Figure S1

Figure S1. *Ror and Rev-erb* expression patterns and *Rorc–/–* mouse behavioral rhythms.

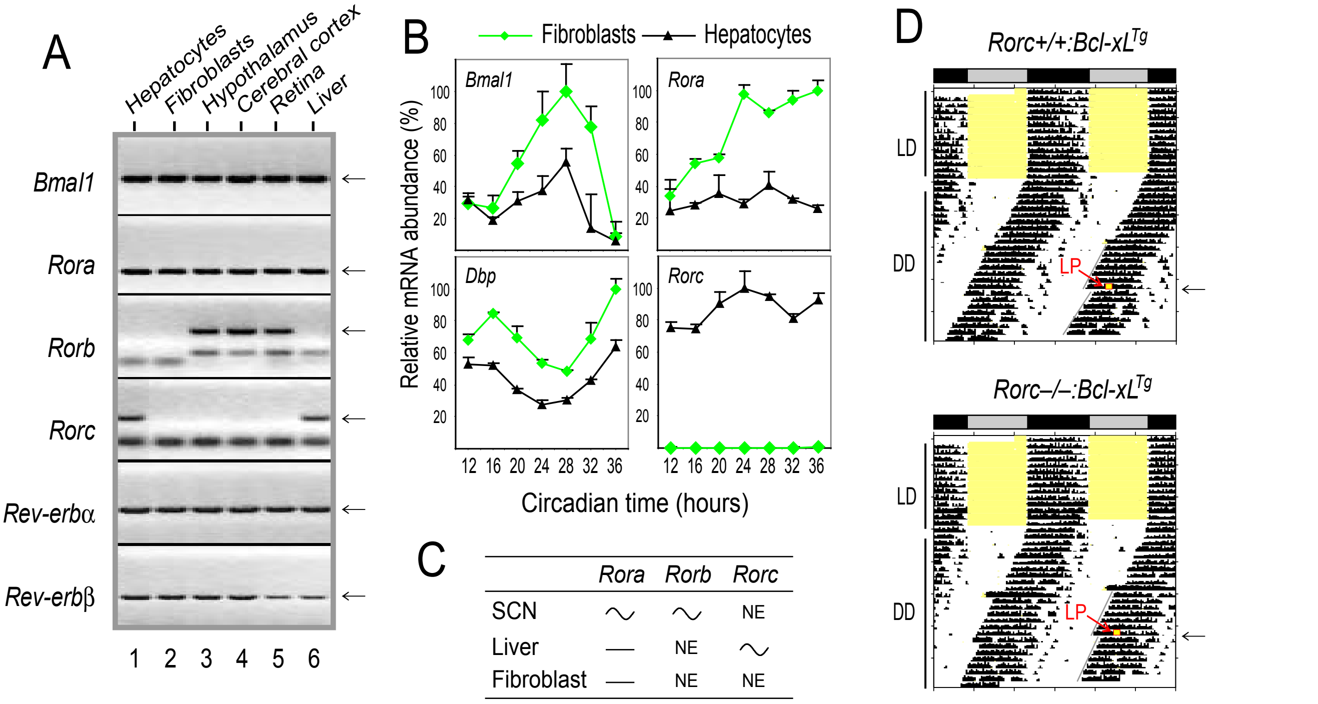

(A) Tissue-specific expression of the *Ror* and *Rev-erb* genes. Total RNA was isolated from the tissues indicated, and gene expression was determined by standard reverse transcription and PCR (RT-PCR) followed by agarose gel electrophoresis.

(B) Temporal mRNA expression profiles of *Bmal1*, *Dbp*, *Rora* and *Rorc* in wild-type fibroblasts and hepatocytes. Expression was analyzed at 4-hr intervals by quantitative PCR (Q-PCR). Values are expressed as percentage of maximum expression for each gene. Error bars represent standard deviation (SD) of expression levels from two culture samples. Circadian time: hours after serum treatment.

(C) Summary of *Rora*, *Rorb* and *Rorc* expression in the SCN, liver, and fibroblasts. Curved line: rhythmic expression. Flat line: arrhythmic expression. NE: not expressed or expression not detected. Note that in fibroblasts, *Rorb* and *Rorc* are not detected, and *Rora* expression is arrhythmic. *Rorc* is not expressed in the SCN, but is rhythmically expressed in liver.

(D) Double-plot actograms for $Bcl\text{-}xL^{Tg}$ controls and homozygous $Rorc^{-/-}:Bcl\text{-}xL^{Tg}$ mice. $Rorc^{-/-}$ mice displayed normal circadian locomotor activity under constant darkness and normal phase shifts in response to a light pulse, compared to controls. Yellow shading represents the light period of LD cycles. Red arrows indicate a light pulse applied at CT16.
