# Supplementary material for: Redundant Function of REV-ERBα and β and Non-Essential Role for Bmal1 Cycling in Transcriptional Regulation of Intracellular Circadian Rhythms
Source: PLoS Genet. 2008 Feb 29;4(2):e1000023. doi: 10.1371/journal.pgen.1000023 (PMC2265523; doi:10.1371/journal.pgen.1000023)
Supplement: Text S1 — Supplemental Materials and Methods. (A) Expression vector construction. (B) shRNA vector construction. (C) TaqMan PCR primers and probes used in this study. (0.10 MB PDF) [file pgen.1000023.s002.pdf]

Liu AC, et al., *PLoS Genetic*, 2008
"Redundant Function of REV-ERBα and β and Non-Essential Role for *Bmal1* Cycling in Transcriptional Regulation of Intracellular Circadian Rhythms"

Supporting Information
Supplemental Text S1

Text S1: Supplemental Materials and Methods

(A) Expression Vector Construction:

Unless otherwise indicated, all cDNA sequences in pENTR/D-cDNA vectors were subsequently cloned into pLenti6/R4R2/V5-DEST destination vector (Invitrogen) in MultiSite Gateway recombination reactions with pENTR-5'-promoter plasmid to generate pLenti6-B4B2 expression constructs as follows.

<u>pENTR-5' promoter constructs:</u>

- Per2 promoter: The promoter DNA fragment of 526 bp was amplified from a mouse BAC clone containing Per2 gene using a forward primer (5'-CTCGAGCGGATTACCGAGGCT GGTCACGTC-3') and a reverse primer (5'-CTCGAGTCCCTTGCTCGGCCCGTCACTT GG-3'), and cloned into pENTR-5'-TOPO vector.
- Bmal1(WT) or Bmal1 promoter: The promoter DNA fragment of 550 bp was amplified from a mouse BAC clone containing Bmal1 gene using a forward primer (5'-GATCGAG CTCGCAGAGTCCGCAACGCAGT GG-3') and a reverse primer (5'-GATCCTCGAGCG CACCCGCACTCGGATCCCG-3'), and cloned into pENTR-5'-TOPO vector.
- Bmal1(Mut) promoter: It is identical to Bmal1(WT) promoter except that the two RORE sites were mutated from (5'-AAAGTAGGTTA---AAAGTAGGTCA-3') to (5'-AAAGTCCT AGC---AAAGTCCTAGC-3').
- UbC promoter: The vector was purchased from Invitrogen.
- CAG promoter: The promoter DNA fragment was digested with Cla I and Bam HI from pLV156-RRLsinPPT-CAG-EGFP-PRE vector DNA (a gift from Inder Verma at the Salk Institute) and cloned into pENTR-5' by replacement of a pre-existing Cla I and Bam HI DNA fragment.

<u>pENTR/D-cDNA constructs:</u>

- dLuc: The dLuc contained a *luciferase* gene and a PEST sequence for rapid protein degradation. The *dLuc* DNA fragment was amplified by PCR and cloned into pENTR/D-TOPO vector.
- Bmal1-Flag: The Flag DNA sequence (5'-GACTACAAGGACGACGATGACAAG-3') was first incorporated into Bmal1 cDNA at the 3'-end. The Bmal1-Flag DNA fragment was then amplified using a forward primer (5'-<u>cacc</u>AAGCTTCCACCATGGCGGACCAGAGA -3') and a reverse primer (5'-GGATCCCTACTTGTC ATCGTCGTCCT-3'), and cloned into pENTR/D-TOPO vector. The nucleotides <u>cacc</u> was added for cloning into pENTR/D-TOPO vector.
- Bmal1::Luc: The Luc DNA fragment was first amplified from pGL3-Basic (Promega) using a forward primer (5'-<u>cacc</u>ATGGAAGACGCCAAAAACATAAAG-3') and a reverse primer (5'--3') and cloned into pENTR/D-TOPO vector which contains an Asc I site at 3'-end. This vector was used as a template to amplify the Luc DNA fragment again using a forward primer containing Asc I restriction site (5'-TG<u>GGCGCGCC</u>ATGGAAGACGCCAAAAACATAAAG-3') and a reverse primer (5'-AACTTTGTACAAG AAAGCTGG-3'), resulting in a DNA fragment containing Asc I sites on both ends, which was then cloned in pCR2.1-TOPO vector. An Asc I restriction fragment from the above vector was subsequently inserted to pENTR/D-Bmal1 vector at the Asc I site to generate pENTR/D-Bmal1::Luc fusion construct.
- Rev-erbα: The Rev-erbα DNA fragment was amplified using a forward primer (5'-<u>cacc</u>AAGCTTCCACC ATGGGGACGACCCTGGACTCCAAT-3') and a reverse primer (5'-TTTCTGGGCGTCCACCCGGAAGGA-3'), and cloned into pENTR/D-TOPO vector.
- Rev-erbβ: The Rev-erbβ DNA fragment was amplified using a forward primer (5'-<u>cacc</u>ATGGAGCTGAA CGCAGGAG-3') and a reverse primer (5'-AGGATGAACTTTAAAGGCC-3'), and cloned into pENTR/D-TOPO vector.

pLenti6-B4B2 expression constructs:

All these constructs contain Blasticidin resistance gene. Cell can be selected with 10 μg/ml Blasticidin following infection.

- Per2-dLuc reporter
- Bmal1-dLuc reporter
- Bmal1(WT)-dLuc: same as Bmal1-dLuc, for comparison with Bmal1(Mut)-dLuc
- Bmal1(Mut)-dLuc
- UbC-dLuc
- Bmal1(WT)-Bmal1-Flag
- Bmal1(Mut)- Bmal1-Flag
- UbC-Bmal1-Flag
- Bmal1(WT)-Bmal1::Luc
- Bmal1(Mut)- Bmal1::Luc
- UbC-Bmal1::Luc
- CAG-Rev-erbα
- CAG-Rev-erbβ

(B) shRNA Vector Construction:

To generate shRNA-expressing constructs against Rev-erbβ, oligonucleotide (nt) sequences were designed using Sfold (sfold.wadsworth.org) and Invitrogen's BLOCK-iT RNAi designer (rnaidesigner.invitrogen.com). Each oligonucleotide contains a sense strand and an antisense strand of 20- or 21-nt, separated by a 4- or 9-nt hairpin loop (underlined). The nucleotides cacc on the top strand and aaaa on the bottom strand (lower case, underlined) were added for cloning into pENTR/U6 vector. U6-shRNA was subsequently cloned into the pLL3.7GW vector. Expression of siRNA was achieved by transcription of shRNA driven by an RNA polymerase III promoter. Infected cells were sorted by FACS for the highest 10% GFP-expressing cells, which represent the highest shRNA-expressing cells. Listed below are targeted oligonucleotide sequences used to generate the three functional shRNA constructs:

shRNA-β1 corresponding to nt 1430-1450 of Rev-erbβ:
5'-caccGGGATCTGCTCAGCTCTATGTCGAAACATAGAGCTGAGCAGATCCC-3' (top strand)
5'-aaaaGGGATCTGCTCAGCTCTATGTTTCGACATAGAGCTGAGCAGATCCC-3' (bottom strand)

shRNA-β2 corresponding to nt 1594-1614 of Rev-erbβ:
5'-caccGCACTAAGGACCTTAATAATGCGAACATTATTAAGGTCCTTAGTGC-3' (top strand)
5'-aaaaGCACTAAGGACCTTAATAATGTTCGCATTATTAAGGTCCTTAGTGC-3' (bottom strand)

shRNA-β3 corresponding to nt 552-571 of Rev-erbβ:
5'-caccGACAGAGAATGCTAATTGAATTCAAGAGATTCAATTAGCATTCTCTG-3' (top strand)
5'-aaaaCAGAGAATGCTAATTGAATCTCTTGAATTCAATTAGCATTCTCTGTC-3' (bottom strand)

(C) TaqMan PCR Primers and Probes Used in This Study:

Gapdh forward:	5′-TGTGTCCGTCGTGGATCTGA-3′
Gapdh reverse:	5′-CCTGCTTCACCACCTTCTTGA-3′
Gapdh probe:	5′-FAM-CCGCCTGGAGAAACCTGCCAAGTATG-BHQ-3′
Per1 forward:	5′-AAACCTCTGGCTGTTCCTACCA-3′
Per1 reverse:	5′-AATGTTGCAGCTCTCCAAATACC-3′
Per1 probe:	5′-FAM-ATCAACTGCCTGGACAGCATCCTC-BHQ-3′
Per2 forward:	5′-CTGGCTCAGCCCTGTCTAGAA-3′
Per2 reverse:	5′-AGTCAATGCTTCCAAAGTATTTGCT-3′
Per2 probe:	5′-FAM-CATCCGCCACCTCAGACTCTCTGGG-BHQ-3′
Cry1 forward:	5′-CGGTTGCCTGTTTCCTGACT-3′
Cry1 reverse:	5′-GCTCCAATCTGCATCAAGCA-3′

Cry1 probe: 5′-FAM-TCCCAGCTGATCCACAGGTCACCA-BHQ-3′

Dbp forward: 5′-CCGTGGAGGTGCTAATGACCT-3′
Dbp reverse: 5′-CCTCTGAGAAGCGGTGTCT-3′
Dbp probe: 5′-FAM-TGAACCTGATCCCGCTGATCTCGCC-BHQ-3′

Bmal1 forward: 5′-AGTACGTTTCTCGACACGCAATAG-3′
Bmal1 reverse: 5′-TGTGGTAGATACGCCAAAATAGCT-3′
Bmal1 probe: 5′-FAM-CGCCCTCTGATCTACAAAAACAAATTTCCCA-BHQ-3′

Clock forward: 5′-TTGCTCCACGGGAATCCTT-3′
Clock reverse: 5′-GGAGGGAAAGTGCTCTGTTGTAG-3′
Clock probe: 5′-FAM-ACACAGCTCATCCTCTCTGCTGCCTTTC-BHQ-3′

Npas2 forward: 5′-TGCTCCGAGAATCGAATGTG-3′
Npas2 reverse: 5′-TCAGTGAACGGCCGCTG-3′
Npas2 probe: 5′-FAM-TATCCGCCCAGGGTCCAAAGCC-BHQ-3′

E4bp4 forward: 5′-GCGAGTTTGAAGGCATGCA-3′
E4bp4 reverse: 5′-CCATGTTTCTCCAGGTCAAAATG-3′
E4bp4 probe: 5′-FAM-CTCTCTTCACCCGCCGATGCGAT-BHQ-3′

Revα forward: 5′-CAAGGCAACACCAAGAATGTTC-3′
Revα reverse: 5′-TTCCCAGATCTCCTGCACAGT-3′
Revα probe: 5′-FAM-TGTATCCCCATGGACGCAGCGG-BHQ-3′

Revβ forward: 5′-AGTAGGTGGATGTTCTCAGACTGAGA-3′
Revβ reverse: 5′-ATGGAGACTTGCTCATAGGACACAC-3′
Revβ probe: 5′-FAM-CAGAAATAGTTACCTGTGCAACACTGGAGGGAG-BHQ-3′

Rora forward: 5′-ATGGAGCTGTGTCAAAATGATCA-3′
Rora reverse: 5′-AGGCACGGCACATCCTAATAA-3′
Rora probe: 5′-FAM-CACCACCTCTAGCGAGCCTGCTTTTAGAA-BHQ-3′

Rorc forward: 5′-CACGGCCCTGGTTCTCAT-3′
Rorc reverse: 5′-CAGATGTTCCACTCTCCTCTTCTCT-3′
Rorc probe: 5′-FAM-TGCCAACCGTCCTGGGCTCC-BHQ-3′

Apoc3 forward: 5′-CATCTGCCCGAGCTGAAGAG-3′
Apoc3 reverse: 5′-CTTGTTCCATGTAGCCCTGTAC-3′
Apoc3 probe: 5′-FAM-TAGAGGGATCCTTGCTGCTGGGCTC-BHQ-3′

Fkbp4 forward: 5′-CACTCCGACTGGCCTCACA-3′
Fkbp4 reverse: 5′-CTTTCGATGGCAGCTGAGAAG-3′
Fkbp4 probe: 5′-FAM-ATCTGGCCATGTGTCACCTGAAACTGC-BHQ-3′

Hsp60 forward: 5′-CCATGCTTGGAGATTTTGTGAA-3′
Hsp60 reverse: 5′-CCAGTAAGGCAGTTCTCACAACCT-3′
Hsp60 probe: 5′-FAM-ATGGTGGAAAAAGGGATCATTGATCCAACA-BHQ-3′

Tubb5 forward: 5′-GGCCTCAAGATGGCAGTCA-3′
Tubb5 reverse: 5′-TGCTCAGAGATGCGCTTGAA-3′
Tubb5 probe: 5′-FAM-CTTCATTGGAAACAGCACAGCCATCCA-BHQ-3′

V1a forward: 5′-CCAGATGTGGTCAGTCTGGGATA-3′
V1a reverse: 5′-GCCGTGATCGTGGTGGA-3′
V1a probe: 5′-FAM-TCGTTTGGACCGATTCCGAAAACCC-BHQ-3′
